# Using mobile phone data helps estimate community-level food insecurity: Findings from a multi-year panel study in Nepal

**DOI:** 10.1371/journal.pone.0241791

**Published:** 2020-11-05

**Authors:** Lichen Liang, Robin Shrestha, Shibani Ghosh, Patrick Webb

**Affiliations:** 1 Gerald J. and Dorothy R. Friedman School of Nutrition Science and Policy at Tufts University, Boston, Massachusetts, United States of America; 2 Feed the Future Innovation Lab for Nutrition, Boston, Massachusetts, United States of America; University of Reading, UNITED KINGDOM

## Abstract

Household food insecurity remains a major policy challenge in low-income countries. Identifying accurate measures that are relatively easy to collect has long been an important priority for governments seeking to better understand and fund solutions for communities in remote settings. Conventional approaches based on surveys can be time-consuming and costly, while data derived from satellite imagery represent proxies focused on biological processes (such as rainfall and crop growth) lack granularity in terms of human behaviors. As a result, there has recently been interest in tapping into the large digital footprint offered by mobile phone usage. This paper explores empirical relationships between data relating to mobile phones (ownership and spending on service use), and food insecurity in rural Nepal. The work explores models for estimating community-level food insecurity through aggregated mobile phone variables in a proof-of-concept approach. In addition, sensitivity analyses were performed by considering the performance of the models under different settings. The results suggest that mobile phone variables on ownership and expenditure can be used to estimate food insecurity with reasonable accuracy. This suggests that such an approach can be used in and beyond Nepal as an option for collecting timely food insecurity information, either alone or in combination with conventional approaches.

## Introduction

Food insecurity (FIS) is an important policy concern for many low- and middle-income country (LMIC) governments. People who live in food insecure households typically consume a nutrient-poor diet which contributes to various forms of undernutrition, which in turn is a leading cause of preventable child mortality globally [1–4]. Progress in reducing household food insecurity depends on an ability to monitor food security indicators for high-risk populations in ways that allow governments and their partners to respond quickly through evidence-based programming.

Problem assessment and targeted responses both require access to good data at a reasonably high spatial resolution and temporal frequency [5]. For instance, data on food consumption patterns and livelihoods are commonly obtained through household surveys at relatively high cost, coupled with contextual data relating to agricultural and climatic conditions via satellite imagery [6]. However, such large-scale data collection is challenging or economically infeasible in LMICs with at-risk remote rural populations who are difficult to access. Long distances, a lack of transport infrastructure, and mountainous topography can impair data collection and limit the monitoring of government programs established in response to need. New ways of generating information are critical for expanding efficient programming that responds to food security needs.

Digital communications data offer a relatively new source of information that can be linked with outcomes of interest at country scale and/or down to communities or individuals. Globally, digital technologies have significantly revolutionized information exchange and communication during the last three decades [7]. Integral to this transformation has been the advent of mobile phone technologies that has enabled rapid transmission of information, including in resource constrained settings [8]. Mobile phones have become the most accessible and communication technology, especially for poorer households facing barriers of access to information and long-distance communication [8]. The International Telecommunication Union (ITU) recently reported that the mobile subscription penetration in developing countries was 103 per 100 people 2018 compared with 128 per 100 people in high income economies, while the penetration in least developed countries surged from 5 mobile subscriptions per 100 people in 2005 to 73 per 100 people in 2018 [9].

While access to mobile phones have increased, a disparity exists in its uptake and usage, largely due to socio-demographic factors such as income, age, gender, education and location [10, 11]. However, ongoing liberalization of relatively low infrastructure costs to set up mobile towers and distribute SIM cards in LMICs have significantly lowered the cost of mobile phones and improved digital inclusion [12]. In Nepal, for instance, although less than 35 percent of the population had internet access in 2017 [13], mobile phone subscriptions grew from 0.043 per 100 people in 2000 to 139 per 100 people in 2020 [14]. The latter compares with a global average of just 104 [15]. In other words, the so-called ‘digital divide’ in terms of mobile phone ownership is being bridged very rapidly [16]. As the physical access of mobile phone use becomes more widespread, data on ownership and usage offer a new source of information relating to household demographics and spending choices. For instance, regional aggregated measures of phone penetration and use have been shown to correlate with regionally aggregated population statistics from census and household surveys [17, 18]. In Nepal, the 2016 Nepal Demographic Health Surveys reported widespread distribution of mobile phone ownership by gender (73% of women and 89% of men), and location (87% of the rural and 90% of urban dwellers) [19].

Blumenstock et al. also showed that an individual’s record of mobile phone use can be used to infer socioeconomic status [20]. As mobile phone use scales-up, this offers new potential for data mining to help in amplifying the economic gains of development. Analysis of the extent and nature of access and use of mobile phones will, for example, be important to help understand who is still being left behind [8].

An attempt to link mobile phone data to conditions of food security at the sector level was made in 2014 by a study conducted by the United Nations, which generated a model to determine a proxy indicator for poverty, based on aggregate mobile phone activity data at a scale of 10000–50000 inhabitants [21]. Although the potential exists for mobile phone ownership and expenditure data to contribute to novel food insecurity indicators, there remains a need to test and validate approaches and demonstrate feasibility in real world settings.

Responding to that need, this study seeks to assess the potential for using data on mobile phone ownership and usage to build a proxy indicator of community level food insecurity in Nepal. We used longitudinal household level panel data that are representative at the national level [22]. Included are data on mobile phone usage as well as a range of relevant household and community-specific information relating to food insecurity. This allowed us to explore empirical relationship using mobile phone variables and food insecurity in rural Nepal and to build models that estimate food insecurity at a relatively small level of granularity. We also compared mobile phone indicators with other community indicators as part of a sensitivity analysis to establish the estimation performance of such models.

## Methods

### Study design

In this study, we explore whether access to, and use of, mobile phones can be used to estimate household food security aggregated at a community level. We draw on the Policy and Science for Health, Agriculture and Nutrition (PoSHAN) survey for this study [22]. The multi-year survey used a nationally representative longitudinal panel design to map out pathways through which agriculture may improve maternal and children health and nutrition. Our secondary analysis was approved by the Tufts University Institutional Review Board for Social, Behavioral and Educational Research as IRB Study Number 1606018, and excluded from further review on June 14, 2016. Ethical clearance for primary data collection was obtained from the Institutional Review Boards of Johns Hopkins University (USA) and the Nepal Health Research Council (Kathmandu).

The results presented here derive from annual surveys conducted over four years in the three agroecological (Mountains, Hills and Terai) regions of Nepal. Using a systematic random sampling, 21 Village development committees (VDCs) were selected (7 VDCs from each agroecological region), in which 63 wards (3 out of total 9 wards per VDC) were visited and all eligible households with children under the age of 60 months were included in the study. That third stage at the ward level included enrolment of households. Around 5,000 households were repeatedly visited during the same season between 2013 and 2016. In each annual survey, additional eligible households were tracked and enrolled to the study, making a note of emigrant households that no longer were eligible due to lack of eligible children (less than 60 months), or aging out of previously eligible children. The massive earthquake in 2015 resulted in a truncated sample (wards n = 27). Data were collected at the community, household and individual levels and included domains of household food security, household socio-economic and demographics, agriculture practices, access to markets, communications and infrastructure, water, hygiene and sanitation, food consumption/production patterns and health, diet and anthropometry of women of reproductive age and children under five by anthropometry. Data on socio-economic status and household assets, household food security, economic shocks in the past year were collected from heads of household. Specific details on survey design, sampling strategy, data collection and management are published elsewhere [22, 23].

### Data description

#### Community selection

The purpose of this study was to estimate community-level food insecurity using mobile phone use variables as reported by households in those communities. In Nepal, the lowest administrative unit is the ward, which incorporated about 100 families. In this study, we used the ward as ‘a community’ because it was large enough to derive community-level (collective use) indicators but small enough to have an adequate sample size for analysis.

#### Community level mobile phone variables: Ownership and expenditure

The mobile phone data were derived from the survey data itself. There were two mobile phone variables in the PoSHAN survey data, namely (1) mobile phone ownership and (2) mobile phone monthly usage (service top-up) expenditure; both were recorded at the household level. Two community-level mobile phone variables were additionally constructed: (1) mobile phone ownership, that is the average number of mobile phones owned by households aggregated at community level, and (2) mobile phone expenditure, that is the average monthly mobile phone expenditure of all households in that community.

#### Community food insecurity status indicators

To assess food insecurity, the PoSHAN survey data used the validated Household Food Insecurity Access Scale (HFIAS) [24]. HFIAS uses self-reported behaviors and perceptions of food insecurity through a set of nine questions administered to one or two adults in a household (usually the senior woman responsible for food) [25]. The responses for each HFIAS question were utilized to compute an HFIAS score and a level of household food insecurity. HFIAS score was a continuous variable, ranged from 0 to 27, with a higher score indicating higher household food insecurity. The levels of household food insecurity were categorized as none, mildly, moderately, and severely food insecure (for more details see Table 4 in [25]).

We aggregated data at the ward level and constructed two community-level indicators to measure food insecurity: (1) FIS score, that is the average of the HFIAS scores of all households in the community; (2) FIS prevalence, that is the percentage of food insecure (including mildly, moderately, and severely food insecure) households in the community.

#### Community socioeconomic status variable

The association between socioeconomic status and household food security was well established [24]. Following the approach used by the Nepal Demographic and Heath Surveys, we constructed a socioeconomic status (SES) indicator (wealth index) for each household using the number and kinds of goods that household own (television, bicycle, etc.), housing characteristics such as access to drinking water, toilet facilities, and flooring materials. The households were categorized as poorest, poorer, middle, richer, and richest [19]. The community socioeconomic status variable was the median SES status of all households in the community.

#### Agro-ecological region variable

Nepal consists of three major agro-ecological regions: the mountains, hills and the plains bordering India (also called the Terai). These regions run broadly parallel from east to west from north to south. There are significant variations among these three regions in terms of climate, biogeography, resources, infrastructure and socioeconomic development. The variation in food security in Nepal by agro-ecological region has been well documented [26, 27], therefore, we included a variable for the agro-ecological region.

#### Analytical strategy

This study was community-level analysis, thus all indicators represented community level indicators, including mobile phone ownership, mobile phone expenditure, FIS score, FIS prevalence, socio-economic status. Descriptive statistics were computed for all the indicators used in the analysis, and annual differences were assessed using ANOVA test or Chi-Square test when applicable. Mobile phone variables and food insecurity indicators were also compared across socioeconomic status.

To capture the empirical relationship between mobile phone ownership/expenditure and food insecurity, given the structure of the panel data, we calculated first-differences between survey rounds in order to document period changes of mobile phone ownership and expenditure as well as fluctuations in food security indicators. Scatterplots were used to illustrate the relationship between period-on-period mobile phone ownership/expenditure vs. period-on-period food insecurity.

We built predictive models using variables of mobile phone usage to estimate community food insecurity. Due to the nature of the survey sampling design, FIS measures were nested within individual ward which were nested within VDC. To account for any interdependence among observations within each level of clustering, multilevel (mixed) models [28] were used. Multilevel modeling is a statistical technique designed to facilitate inferences from hierarchical data as well as a powerful tool for generating predictions [29]. The levels of clustering in the multilevel model were specified as random effects while the independent variables were specified as fixed effects. The predictive model is formulated as:

$$Y_{ijk}=\max\left(0,\alpha_{0}+\beta X_{ijk}+u_{jk}+v_{k}+\in_{ijk}\right)$$

where *i* = 1,‥4 *panels*, *j* = 1,…63 *wards*,*k* = 1,…21 *VDCs*. *Y*_*ijk*_ represents FIS measures, *α*_0_ refers to grand mean across all VDCs, $v_{k}\sim N(0,\sigma_{v}^{2})$ models variation between VDC, $u_{jk}\sim N\left(0,\sigma_{u}^{2}\right)$ models variation between wards within a VDC, $\epsilon_{ijk}\sim N\left(0,\sigma_{\epsilon }^{2}\right)$ represents residual errors not accounted by the model. ***X***_*ijk*_ refers to a vector of predictors, which were selected from a set of predictors, including mobile phone ownership (MO), mobile phone expenditure (ME), argo-ecological region (REGION), socio-economic status (SES), and survey panel year (YEAR).

Various model specifications were constructed to examine model estimation performance in different settings and compare mobile phone predictors with other predictors. Maximum restricted likelihood was used to estimate model parameters. Operator max(0, *y*) ensures that the output is non-negative since FIS measures are always non-negative. S1 Appendix provides in-depth details on the model specifications used in this analysis.

The model performance was evaluated using the mean absolute error (MAE), which is the average of the absolute difference between the estimated FIS measure and the observed FIS measure. We also calculated the standard deviation of the absolute error (SDAE) which can characterize the dispersion of errors. The smaller the MAE and SDAE, the better the model. We calculated the error (MAE and SDAE) on the data, called the in-sample error. A caveat of using the in-sample error is that it exaggerates how well it will do out of sample. To further characterize model performance in practice, we conducted cross-validation [30]. Because of the structure of the panel data, we cross-validated stratified by either VDC or panel year, which simulated two different practical scenarios. In the cross-validation stratified by VDC, we reserved one VDC sample as test and used other 20 VDC samples to train the model. We repeated this cross validation 21 times such that each VDC served as a test sample once. This simulated a scenario of estimating FIS of wards and VDCs that were not in part of our survey. We also ran cross-validations stratified by panel, where the data from panel 1–3 were reserved for training, and tested the model using the data from panel 4. This allowed us to simulate a scenario of assessing current food insecurity status from FIS measures of the past few years. All statistical analysis was carried out using STATA (version 14.0, College Station, TX).

## Results

The aggregated dataset has 215 ward-level observations; panel 1, 2 (2013 and 2014) included 63 wards while panel 3 in 2015 included 27 wards, and panel 4 in 2016 included 62 wards. Out of the total 63 wards enrolled in the study, 27 wards (43%) were followed up in all four panel (2013–2016), 35 wards (55.6%) were followed up in three panel (2013, 2014 and 2016), 2016), and one ward was followed up across two panels (2013 and 2014). Table 1 provides the descriptive of the community variables used in the analysis. Ward-level food insecurity varied significantly across all four panels (p<0.001). There were statistically significant differences in mean mobile phone ownership across panels (p<0.001). Mobile phone expenditure did not show significant variation across panel years (p = 0.52). The distribution of samples across the three regions varied across years, while the sample distribution by socioeconomic status did not differ significantly across years. We found that both mobile phone ownership and mobile phone expenditure increased from 2013 through 2016, which is in line with demand growth for mobile phones in many LMICs [31]. Food security also improved over the years.

**Table 1:** Descriptive statistics of community-level indicators, by panel/survey year.

	All panels (n = 215)	Panel 1 (2013) (n = 63)	Panel 2 (2014) (n = 63)	Panel 3 (2015) (n = 27)	Panel 4(2016) (n = 62)	p-value*
FIS score	1.18 (0.54,2.72)	2.24(1.19,3.64)	1.30 (0.58,3.01)	0.55(0.33,1.01)	0.82 (0.36,1.54)	<0.001
FIS prevalence	0.28(0.14,0.49)	0.42(0.27,0.58)	0.29(0.16,0.50)	0.16(0.08,0.28)	0.18(0.08,0.38)	<0.001
Mobile phone ownership	1.77(1.43,2.05)	1.67(1.19,1.89)	1.73(1.33,1.93)	1.90(1.59,2.17)	2.03(1.65,2.23)	<0.001
Mobile phone expenditure (Nepalese Rupees)	566 (391,757)	470 (340,720)	554 (405,754)	642 (482,803)	613 (438,814)	0.519
Argo-ecological regions						
Mountains	30.2%	33.3%	33.3%	11.1%	32.2%	0.003
Hills	30.7%	33.3%	33.3%	11.1%	33.9%	
Terai	39.1%	33.3%	33.3%	77.8%	33.9%	
Socioeconomic status						
Poorest	12.1%	14.3%	14.3%	7.4%	9.7%	0.931
Poorer	34.9%	31.7%	38.1%	37.0%	33.9%	
Middle	28.8%	30.2%	23.8%	29.6%	32.3%	
Richer	18.1%	17.5%	15.9%	26.0%	17.7%	
Richest	6.1%	6.3%	7.9%	0%	6.4%	

Note: Values for the continuous variables provided as medians (25th,75th percentiles in parentheses). Values for the categorical variables provided as percentage.

*p-values obtained by Chi-square or ANOVA tests, where applicable.

S1 Table shows the coefficient matrix of community mobile phone variables (MO and ME) and measures of community food insecurity (FIS score and FIS prevalence). Correlation between two measures of food insecurity was very high (*r* = 0.902, p<0.001). The two mobile phone variables (MO and ME) were also highly correlated (*r* = 0.665, p<0.001). High correlations were also observed between mobile phone ownership and food insecurity measures (with FIS score, *r* = -0.619, p<0.001; with FIS prevalence, *r* = -0.583, p<0.001), while significant, modest correlations were observed between mobile expenditure and food insecurity measures (with FIS score *r* = -0.342, p<0.001, with FIS prevalence *r* = -0.349, p<0.001).

Table 2 presents the descriptive statistics of community food insecurity indicators and mobile phone variables by socioeconomic status. There were statistically significant differences in mean values of all variables (FIS score, FIS prevalence, MO, and ME) by socioeconomic status. That is, as anticipated, wealthier wards tended to have lower levels of food insecurity, own more mobile phones per household, and spend more on mobile phone usage.

**Table 2:** Community food insecurity indicators and mobile phone variables, by socioeconomic status.

	Poorest (n = 26)	Poorer (n = 75)	Middle (n = 62)	Richer (n = 39)	Richest (n = 13)	p-value*
FIS score	3.26 (1.75,5.45)	1.56 (0.79,3.20)	1.23 (0.52,2.31)	0.73 (0.33,1.09)	0.43 (0.41,0.88)	<0.001
FIS prevalence	0.53 (0.40,0.73)	0.33 (0.19,0.51)	0.28(0.14,0.45)	0.16 (0.09,0.23)	0.13(0.10,0.19)	<0.001
Mobile phones ownership	1.30 (1.03,1.59)	1.59 (1.29,1.84)	1.85 (1.52,2.04)	2.03 (1.89,2.21)	2.17 (2.03,2.42)	<0.001
Mobile phone expenditure (Nepalese Rupees)	346 (288,451)	470 (343,571)	683 (448,807)	773 (645,969)	952 (754,1124)	<0.0011

Note: Values for the continuous variables provided as medians (25th,75th percentiles in parentheses).

*p-values obtained by ANOVA tests. The statistics are based on the pooled sample comprising observations from across four panel rounds.

**Fig 1:**
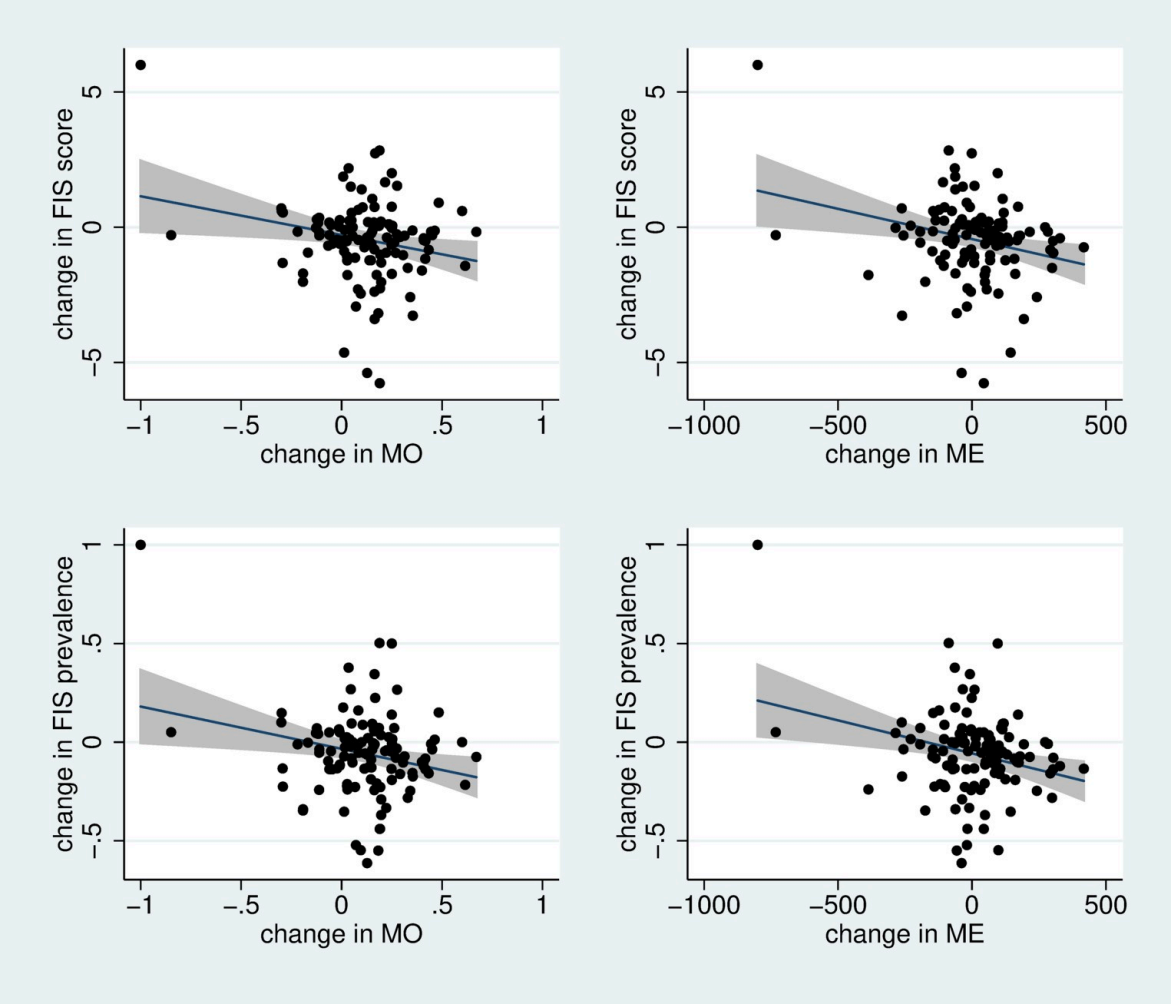
Scatter graphs between period-on-period changes in mobile phone ownership/expenditure and the period-on-period changes in food insecurity. Note: Each mobile phone variable is plotted for each food insecurity measure. The OLS regression line with 95% confidence interval is superimposed on the scatter plot. It is observed that there are negative correlations between the mobile phone variables and the food insecurity measures. ME: mobile phone expenditure; MO: Mobile phone ownership.

Fig 1 illustrates the empirical relationship between period-on-period changes in mobile phone ownership /expenditure and period-on-period changes in food insecurity. All the graphs show negative correlations, indicating that when food insecurity increased, mobile phone ownership/expenditure tended to decrease, which provides an empirical evidence for the potential ability of mobile phone ownership/expenditure to estimate food insecurity.

To examine model estimation performance in different settings as well as to compare mobile phone variables with other predictors, we compared 7 models that include different ranges of predictors: (1) ME only; (2) MO only; (3) SES only; (4) Region only; (5) SES and Region; (6) ME, MO and Region; (7) ME, MO, SES and Region. Models 1–4 were to compare 4 predictors (i.e. ME, MO, SES and Region) when estimating food insecurity separately. Models 5–7 further compared mobile phone variables with SES. All models were multilevel models controlling for multi-level clustering with survey year as a fixed effect.

Tables 3 and 4 show the coefficients of the multilevel linear regression models for FIS score and FIS prevalence, respectively. Although our models were used for prediction, not for inferring any causality, the estimated coefficients still showed some valuable information. We noticed that the coefficients of MO and ME were both negative, which indicated that our model tends to predict lower FIS when mobile phone usage is high. Note that these estimated coefficients were not used for inference, because the endogeneity issue made these estimates biased and inconsistent, which invalidated inferences. These estimated coefficients were only used to obtain good predictions.

**Table 3:** Coefficients of multi-level models estimating FIS score.

	(1)	(2)	(3)	(4)	(5)	(6)	(7)
ME	-0.00230***					-0.000497	-0.000259
MO		-2.103***				-1.863***	-1.757***
Year 2013	0	0	0	0	0	0	0
Year 2014	-0.770***	-0.518**	-0.841***	-0.815***	-0.842***	-0.542**	-0.573**
Year 2015	-1.150***	-0.688*	-1.410***	-1.287***	-1.361***	-0.663*	-0.740**
Year 2016	-1.420***	-0.693**	-1.566***	-1.611***	-1.564***	-0.754**	-0.802***
SES (1–5)			-0.565***		-0.567***		-0.228
Region (Mountains)				0	0	0	0
Region (Hills)				-1.018	-0.907*	-0.692	-0.645
Region (Terai)				-1.327*	-1.187**	-1.337**	-1.249**
Constant	4.140***	6.031***	4.333***	3.591***	5.038***	6.628***	6.898***
Random effects							
Between VDCs	1.093	0.576	0.614	0.869	0.411	0.377	0.312
Between Wards	0.423	0.272	0.297	0.559	0.297	0.282	0.247
Residual	1.238	1.204	1.453	1.383	1.452	1.196	1.213
N	215	215	215	215	215	215	215

Notes: ME: mobile phone expenditure; MO: Mobile phone ownership; SES: socioeconomic status. We assign scores 1,2,3,4, and 5 to the five levels of SES, and treat SES as continuous variable in the model. VDC: Village Development Committee. Estimates obtained using multilevel linear models. Models: (1) y ~ ME; (2) y~MO; (3) y~SES; (4) y~Region; (5) y~Region+SES; (6) y~MO+ME+Region; (7)y~MO+ME+Region+SES. Significance,

* p < 0.05,

** p < 0.01,

*** p < 0.001.

**Table 4:** Coefficients of multi-level models estimating FIS prevalence.

	(1)	(2)	(3)	(4)	(5)	(6)	(7)
ME	-0.000196***					-0.00000154	0.0000265
MO		-0.209***				-0.210***	-0.196***
Year (2013)	0	0	0	0	0	0	0
Year (2014)	-0.0850**	-0.0593*	-0.0918**	-0.0888**	-0.0918**	-0.0591*	-0.0631*
Year (2015)	-0.170***	-0.121**	-0.194***	-0.179***	-0.187***	-0.113**	-0.124**
Year (2016)	-0.174***	-0.0988**	-0.186***	-0.190***	-0.185***	-0.0980**	-0.104**
SES (1–5)			-0.0617***		-0.0617***		-0.0301
Region (Mountains)				0	0	0	0
Region (Hills)				-0.105	-0.0921	-0.0623	-0.0565
Region (Terai)				-0.137	-0.121*	-0.129*	-0.118*
Constant	0.548***	0.756***	0.601***	0.515***	0.672***	0.822***	0.859***
Random effects							
Between VDCs	0.0161	0.0107	0.0112	0.0189	0.0097	0.0090	0.0069
Between Wards	0.0039	0.0014	0.0003	0.0040	0.0003	0.0015	0.0005
Residual	0.0229	0.0228	0.0262	0.0238	0.0262	0.0228	0.0237
N	215	215	215	215	215	215	215

Notes: ME: mobile phone expenditure; MO: Mobile phone ownership; SES: socioeconomic status. We assign scores 1,2,3,4, and 5 to the five levels of SES, and treat SES as continuous variable in the model; VDC: Village Development Committee. Estimates obtained using multilevel linear models. Models: (1) y ~ ME; (2) y~MO; (3) y~SES; (4) y~Region; (5) y~Region+SES; (6) y~MO+ME+Region; (7)y~MO+ME+Region+SES. Significance,

* p < 0.05,

** p < 0.01,

*** p < 0.001.

Table 5 shows in sample errors, and Table 6 show the cross-validation errors, both reporting the mean and standard deviation of absolute errors. All CV errors were larger than corresponding in sample errors, which was anticipated. While in sample error tends to underestimate real error, CV error may overestimate real error since CV only use part of samples to build models. Thus, in-sample error and CV error can tell us a rough range of what the real error would be. We also noticed that the difference between CV errors and in sample errors were small, indicating our models did not have overfitting issues. The best model (7) included all predictors, namely ME, MO, SES, and Region. With this model (7), the MAE for estimating FIS score were 1.013 (in sample) and 1.080 (CV), and the MAE for estimating FIS prevalence were 0.135 (in sample) and 0.148 (CV). Comparing models 1–4, we found that working alone, mobile phone ownership had better performance than SES (MAE(CV) 1.134 vs. 1.135) in estimating FIS score, and SES was better than agro-ecological region (MAE(CV) 1.306) and mobile phone expenditure (MAE(CV) 1.276).) In estimating FIS prevalence, the performance of model with MO was similar to model with SES (MAE, 0.155 vs. 0.153), but SDAE of MO model was much smaller than that of SES model. Mobile phone expenditure provided moderate prediction power, better than the agro-ecological region variable (MAE in FIS score, 1.276 vs. 1.306, MAE in FIS prevalence 0.170 vs 0.178). When SES was not available for the community, Model (6) could be used instead, which still performed well in estimating food insecurity, with MAE of 1.024~1.097 in FIS score, and MAE of 0.140~0.153 in FIS prevalence.

**Table 5:** In sample errors of models estimating community food insecurity.

FIS measure	Model (1)	Model (2)	Model (3)	Model (4)	Model (5)	Model (6)	Model (7)
FIS score	1.224±1.031	1.097±0.867	1.098±1.018	1.198±1.059	1.062±0.935	1.024±0.808	1.013±0.775
FIS prevalence	0.161±0.120	0.149±0.106	0.146±0.121	0.163±0.126	0.141±0.117	0.140±0.105	0.135±0.103

Note: All models are multilevel models. mean±SD of absolute errors between observed and predicted value are reported. Models: (1) y ~ ME; (2) y~MO; (3) y~SES; (4) y~Region; (5) y~Region+SES; (6) y~MO+ME+Region; (7)y~MO+ME+Region+SES. ME: mobile phone expenditure; MO: Mobile phone ownership; y represents food insecurity measure.

**Table 6:** Cross-validation errors of models estimating community food insecurity.

FIS measure	Model (1)	Model (2)	Model (3)	Model (4)	Model (5)	Model (6)	Model (7)
FIS score	1.276±1.071	1.134±0.911	1.135±1.058	1.306±1.126	1.129±1.003	1.097±0.889	1.080±0.865
FIS prevalence	0.170±0.124	0.155±0.112	0.153±0.126	0.178±0.136	0.153±0.126	0.153±0.115	0.148±0.114

Note: All models are multilevel models. mean±SD of absolute errors between observed and predicted value are reported. Models: (1) y ~ ME; (2) y~MO; (3) y~SES; (4) y~Region; (5) y~Region+SES; (6) y~MO+ME+Region; (7)y~MO+ME+Region+SES. ME: mobile phone expenditure; MO: Mobile phone ownership; y represents food insecurity measure.

We asked the question “Are data on mobile phone variables useful to estimate food insecurity?”. The baseline should be a model containing variable for agro-ecological region, that is, model (4), because the region variable is accessible without running a survey. Adding the two mobile phone variables, for FIS score, the model (6) reduced MAE (in sample) from 1.198 to 1.024, and the SDAE from 1.059 to 0.808, which means that the model could increase the accuracy by 14.5% and reduce the dispersion of errors by 21.1%. For FIS prevalence, mobile phone ownership and expenditure variables also helped. Compared with model (4), the MAE (in sample) decreased from 0.163 to 0.140, and the standard deviation decreased from 0.126 to 0.105, reducing errors by 14.1% and the standard deviation by 16.7%. In general, using the mobile phone variables to estimate food insecurity could help reduce errors, which was reflected by smaller MAE and SDAE.

SES is a strong predictor of food insecurity, but SES data are not easily available particularly for remote areas of Nepal. Compared to model (5) that used SES and Region, the model (6) that used the mobile phone variables and Region achieved better estimates. For FIS score, MAE (in sample) of model 5 was 1.062, compared to MAE (in sample) of 1.024 of model 6. For FIS prevalence, model 6 also performed better than model 5 (in sample, MAE 0.140 vs 0.141, SDAE 0.105 vs. 0.117). This comparison shows that, compared with SES, mobile phone variables had stronger predictive power for estimating food insecurity. The above conclusions were still valid when CV errors were used in comparison.

**Fig 2:**
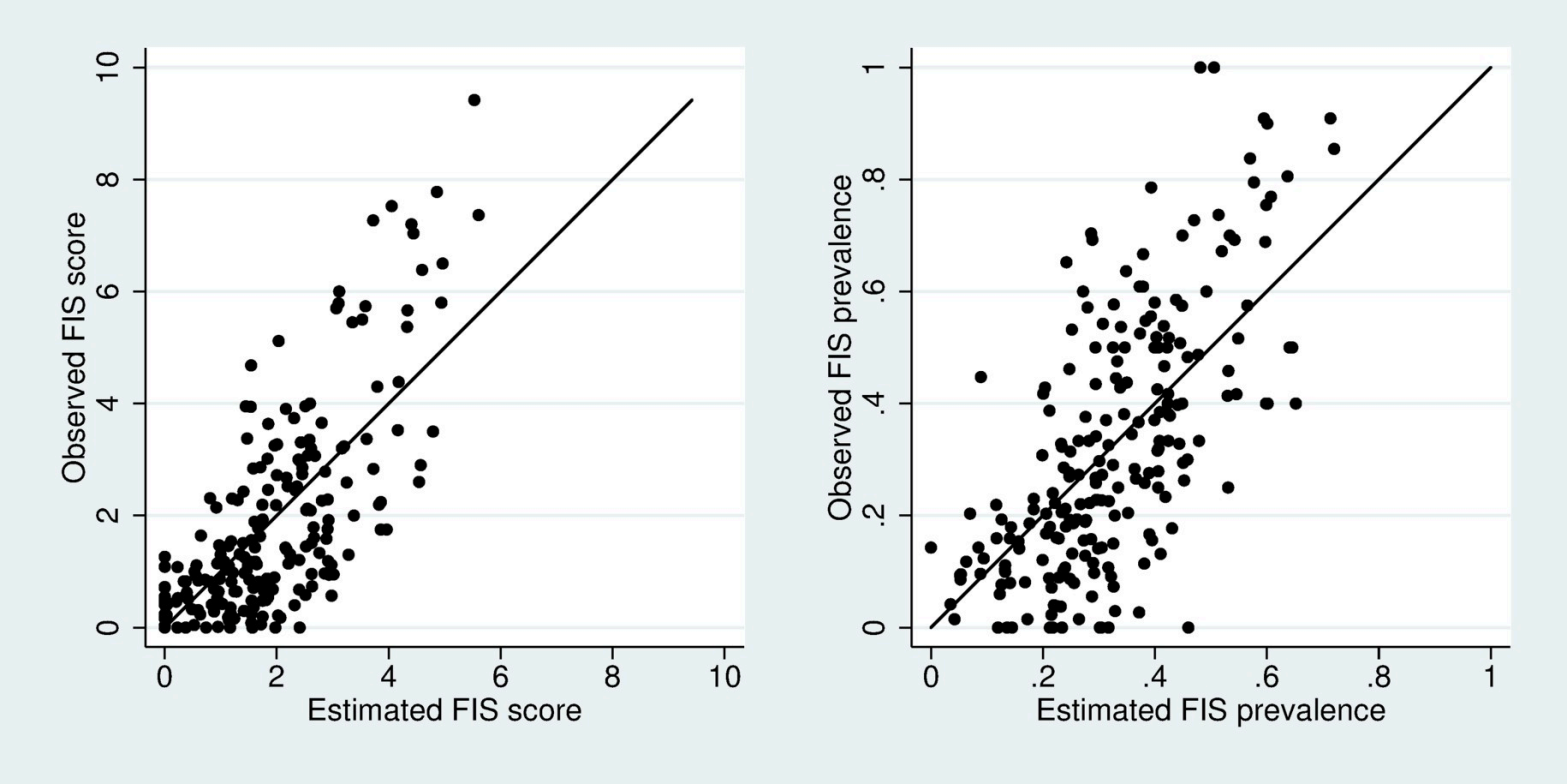
Scatter plots of observed and estimated values for FIS score and FIS prevalence. Note: Estimates are from the model that includes all predictors. The solid line shows the relationship y = x, data points for good models would lie close to this line.

Fig 2 shows the observed and estimated FIS measures with the model containing all predictors. One line shows the relationship y = x. The closer the data are to the line, the better the model, because estimated values are very close to observed values. The average difference between estimated FIS score and the observed values was 1.013, and these estimated values explained 52 percent of variance of the observed FIS scores. The average difference between the estimated FIS prevalence and the observed values was 0.135, and these estimates could explain 46% of variance of the observed FIS prevalence values.

**Fig 3:**
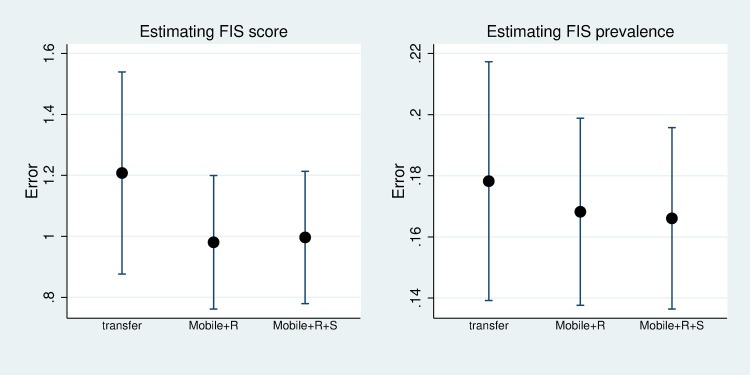
Errors of various approaches estimating FIS of panel 4 from panel 1–3. Note: “transfer method” is compared to two models (mobile+R represents a model including ME, MO, and Region, mobile+R+S represents a model including ME, MO, Region, and SES).

We further tested the performance of the models through cross-validation stratified by the panel. To run this test, the variable YEAR needed to be removed from the model. We trained the model using the data in panels 1–3 and tested it in panel 4. In addition to comparing different model specifications, we also compared a simple method of transferring FIS measures from the previous year to the current year. Fig 3 shows the performances (CV errors) of different models, along with “transfer method”. We found that the performances of models using mobile phone variables were better than the "transfer method". When estimating FIS score, the model using mobile phone had MAE of 0.98, compared to MAE of 1.2 by “transfer method”, indicating an 18% improvement in performance. When estimating FIS prevalence, the MAE of the “transfer method” was 0.178, while the MAE of the model was 0.168, indicating a performance improvement by 5.6%. Incorporating SES into the model helped estimating FIS prevalence but did not help too much for estimating FIS score.

## Discussion

The search for easy-to-collect measures of household food insecurity has long been an important priority for governments and their development partners. Because mobile operators generate very large amounts of mobile phone data, this new source of data can be useful for policy makers to identify and monitor food insecure areas. This study confirms that it is possible to create a viable food insecure proxy indicator using mobile data. With such data, policymakers can timely obtain reasonably accurate food insecurity information without the need to undertake costly and time-consuming surveys in remote regions. This does not imply that models should replace on-the-ground data collection; rather, that remote modeling can complement in-person surveys and potentially improve the targeting of ground-based information gathering to areas of higher risk.

Some attempts have been made to build such tools, demonstrated the potential, and required further research and method validation in other environments. Our research extends previous research in several aspects: (1) this study used a panel data from a nationally-representative household survey in Nepal to study the empirical relationship between mobile phone use and food insecurity; (2) we explored this approach (using mobile phone variables to estimate food insecurity) more thoroughly by examining its use in different situations, including different food insecurity indicators and different model specifications, so as to assess its sensitivity and compare with other predictors.

This study provided empirical evidence for the potential ability of mobile phone ownership/expenditure to estimate food insecurity. Mobile phone variables had high correlation with food insecurity measures, as well as to socio-economic status which is an important determinant factor for food insecurity. More importantly, the panel data allowed us to explore the relationship between period-on-period mobile phone variables and period-on-period food insecurity, which further demonstrated its potential.

There are hypothetical pathways by which mobile phone use may be a viable proxy for household food security. First, there is a literature documenting improved incomes and social networks associated with adoption of mobile phones [10, 32], in part to access information (such as food and other wholesale and retail prices in real time at various competing markets) and services (such as healthcare, access to police or legal support, and having repair and maintenance work done more quickly on, say, irrigation pumps or protected water sources). Second, the widening use of phones across even remote rural areas cements trading relationships that help support active market engagement of smallholders in rural areas [33]. Third, low levels of household food security are associated not only with low income and lack of food stores, they are also driven by physical isolation (remoteness to markets and health centers), social isolation (to lack of community-level social capital that underpins loans and gifts to get over difficult times), and information isolation (low levels of literacy, educational attainment, and exposure to behaviour change messaging linked to health, nutrition, child feeding, etc.). As such, the presence or absence of mobile phones reflect the level of potential interaction the user can have with the wider world. This makes it a strong proxy indicator of ‘security’ relating in this case to food and nutrition.

We explored the possibility of building models that identify food insecure communities using variables that do not rely on large-scale surveys and can be easily accessed by policy makers. Agro-ecological regions and mobile phone variables are such variables. We found that using mobile phone variables greatly improved the estimation of food insecurity, and better performed than a SES indicator in many cases. While SES indicator can be a good representative of food insecurity, it requires data that cannot be obtained without a survey. We examined our results using different measures of food insecurity and conducting cross-validation tests that allowed for a rigorous assessment of model performance. All the conclusions were not sensitive to food insecurity measures and to the cross-validation test. This provided evidence that mobile variables can be used to build such models and may become a new way to measure food insecurity. Due to data limitation, we could only explore two mobile phone variables in this study. As more mobile phone data is available, we expect models with additional mobile phone data to have smaller errors in estimating community-level food insecurity.

There were several practical issues that need to be considered during the model building. First, we needed to consider the context. In Nepal, there are significant variations among these three ecological regions in terms of climate, biogeography, resources, infrastructure and socioeconomic development. Therefore, the models for estimating food insecurity should be adapted to agro-ecological regions. Our results confirmed that models with REGION were better than models only using mobile phone variables. Even if the region factor was incorporated into the model, we still found big variations of model performance across agro-ecological regions. We found that models performed poorly in Mountains region, compared to Hills and Terai regions. Another practical issue was the choice of data aggregation to represent a ‘community’. A smaller geographic granularity is always desired because policy makers can more accurately identify vulnerable groups and can use less resources to act quickly. One problem of analyzing data at the smaller community level is that models are generally less accurate than models at the larger community level [20]. However, working with smaller community can provide more samples for model building, which helps to improve model accuracy. Therefore, there is a trade-off between large community level and large sample size. In this study, we demonstrated that these models can provide moderate food insecurity estimates in relatively small communities (about 100 households).

Mobile phones are becoming more popular in Nepal, and the number of mobile phones owned by households is growing steadily. At the same time, household food security has greatly improved in the past decade. As society is constantly changing, the relationship between mobile phone variables and food insecurity will also change. Our data shows that the correlation between mobile phone variables and food insecurity in 2013–2015 were stable but dropped sharply in 2016, which might be troublesome for models that use past data to predict the future. In this study, we tested the model by using the data of panels 1–3 to estimate FIS panel 4. The performance of the model did not degrade too much and was much better than the simple method of replicating the food insecurity measures of the past year, which provided further evidence to support this approach.

Two food insecurity measures (FIS score and FIS prevalence) were tested in this study to check the sensitivity of the model to selected FIS measures. The two measures were highly correlated, and our models showed consistent results for the two FIS measures. For example, no matter whether FIS score or FIS prevalence was used to measure community FIS, mobile phone variables greatly improved the estimation performance. However, some differences are worth mentioning. After adjusting the survey year and region, the intra-class correlation (ICC) of FIS score was 0.31, and the ICC of FIS prevalence was 0.40, which means that if FIS was measured with FIS prevalence, the wards within a same VDC were more similar than if measured with FIS score, which might affect model performance. Considering the best performance model, the estimated error of FIS score was 1.008 (in sample) ~ 1.013 (CV), and the mean of FIS score was 1.869, indicating the relative error was about 54%. While for estimating FIS prevalence, the estimated error was 0.135 (in sample) ~ 0.148 (CV), and the mean of FIS prevalence was 0.323, indicating the relative error was 42%. In terms of relative error, the model worked better if the community FS was measured by FIS prevalence. Another interesting difference is that the use of SES in combination with mobile phone variables improved the performance more when using FIS prevalence (~3%) than when using FIS score (~1%).

### Limitations

Due to the earthquake in Nepal in 2015, the PoSHAN annual survey for 2015 was truncated, thereby limiting data to nine out of the 21 districts. This reduced the size of the dataset for that survey year. In addition, such analysis ideally would have included call data records (CDRs). Several attempts were made to gather call data records (CDRs) to capture the mobile phone activity of the households from the national mobile phone operators but the researchers were unsuccessful due to data sharing constraints. Thus, CDRs were not included in this analysis.

An additional limitation is linked to the measurement of mobile phone ownership and penetration which has its own challenges. The data used for this analysis were self-reported household recall data that may be confounded by social desirability bias [34]. Another limitation of using mobile phone ownership data is the inability to take into account mobile phone turnovers, such as phones lost, stolen or broken, and dead numbers which may lead to misreporting of actual use and access [8]. Despite the use of only two mobile phone variables and a limited sample size, we are able to present a moderately accurate estimate of food insecurity status at the community level. With more community level indicators available and a bigger sample size, the model performance can be further improved.

While our analysis showed the potential for real-time monitoring of vulnerable groups, one of the challenges for such work is ensuring that there is an agreement that respects the privacy of individual users and the commercial concerns of mobile operators when data is analyzed. Therefore, users are advised to perform spatial aggregation to prevent re-identification of individual users. Our approach to modeling community-level food insecurity can effectively avoid this privacy issue.

## Conclusions

Mobile phones are becoming increasingly popular in low-income country settings. This analysis demonstrated that estimating food insecurity using mobile phone variables is possible, but the ability of such data to aid the real-time monitoring of food security needs to be established in future research using (temporally) more granular data (both on phone use and on food insecurity), and data from network operators. In coming years, the cost-effectiveness of using this approach to implement targeted interventions versus conventional food security assessments should be carefully measured, along with a determination of time frame from assessment to response.

## Supporting information

S1 Dataset Mobile-foodinsecurity. (XLSX)

S1 Appendix Model specifications for all models presented in the main analysis. (DOCX)

S1 Table Correlation coefficient matrix of mobile variables and measures of community food insecurity in Nepal. (DOCX)
