# Supplementary material for: Using mobile phone data helps estimate community-level food insecurity: Findings from a multi-year panel study in Nepal
Source: PLoS One. 2020 Nov 5;15(11):e0241791. doi: 10.1371/journal.pone.0241791 (PMC7644081; doi:10.1371/journal.pone.0241791)
Supplement: S1 Appendix — (DOCX) [file pone.0241791.s002.docx]

# S1 Appendix: Model specifications for all models presented in the main analysis

The data is collected on $n$ wards, and each ward $j=1,..n$ belongs to one of the $k=1,..K$ VDCs. We also have $i=1,..T$ years (panels) in which each $j$ ward is monitored. Here n=63, K=7, and T=4.

A multilevel model framework was constructed.

Level 1: $Y_{ijk}=b_{jk}+\boldsymbol{\beta}\boldsymbol{X}_{\boldsymbol{ijk}}+\epsilon_{ijk}$ where $e_{ijk}\sim N(0,\sigma_{\epsilon}^{2})$

Level 2: $b_{jk}=b_{00k}+u_{jk}$, where $u_{jk}\sim N(0,\sigma_{u}^{2})$

Level 3: $b_{00k}=\alpha_{0}+v_{k}$, where $v_{k}\sim N(0,\sigma_{v}^{2})$

which results in:

$$Y_{ijk}=\alpha_{0}+\boldsymbol{\beta}\boldsymbol{X}_{\boldsymbol{ijk}}\boldsymbol{+}u_{jk}+v_{k}+\epsilon_{ijk}$$

$Y_{ijk}$ refers to the community food insecurity for the ward $j$ nested in VDC $k$ at year $i$.

$\boldsymbol{X}_{\boldsymbol{ijk}}$ refers to a vector of predictors on the level 1.

$b_{jk}$ refers to the intercept in ward $j$, which is nested in VDC $k$.

$\epsilon_{ijk}$ refers to the random errors for the level 1 equation.

$b_{00k}$refers to the overall intercept at VDC k

$\alpha_{0}$refers to the overall intercept across all VDCs.

Based on this multilevel model framework, 7 models were constructed with various predictors.

Model 1. $Y_{ijk}=\alpha_{0}+\beta_{1}{Year}_{ijk}\boldsymbol{+}{\beta_{2}ME_{ijk}+u}_{jk}+v_{k}+\epsilon_{ijk}$

Model 2: $Y_{ijk}=\alpha_{0}+\beta_{1}{Year}_{ijk}\boldsymbol{+}{\beta_{3}MO_{ijk}+u}_{jk}+v_{k}+\epsilon_{ijk}$

Model 3: $Y_{ijk}=\alpha_{0}+\beta_{1}{Year}_{ijk}\boldsymbol{+}{\beta_{5}SES_{ijk}+u}_{jk}+v_{k}+\epsilon_{ijk}$

Model 4: $Y_{ijk}=\alpha_{0}+\beta_{1}{Year}_{ijk}\boldsymbol{+}{\beta_{6}Region_{jk}+u}_{jk}+v_{k}+\epsilon_{ijk}$

Model 5: $Y_{ijk}=\alpha_{0}+\beta_{1}{Year}_{ijk}\boldsymbol{+}\beta_{5}SES_{ijk}+{\beta_{6}Region_{jk}+u}_{jk}+v_{k}+\epsilon_{ijk}$

Model 6: $Y_{ijk}=\alpha_{0}+\beta_{1}{Year}_{ijk}\boldsymbol{+}{\beta_{2}ME_{ijk}+\beta_{3}MO_{ijk}+\beta_{6}Region_{jk}+u}_{jk}+v_{k}+\epsilon_{ijk}$

Model 7:

$Y_{ijk}=\alpha_{0}+\beta_{1}{Year}_{ijk}\boldsymbol{+}{\beta_{2}ME_{ijk}+\beta_{3}MO_{ijk}+\beta_{5}SES_{ijk}+\beta_{6}Region_{jk}+u}_{jk}+v_{k}+\epsilon_{ijk}$
