# Supplementary material for: Using mobile phone data helps estimate community-level food insecurity: Findings from a multi-year panel study in Nepal
Source: PLoS One. 2020 Nov 5;15(11):e0241791. doi: 10.1371/journal.pone.0241791 (PMC7644081; doi:10.1371/journal.pone.0241791)
Supplement: S1 Table — (DOCX) [file pone.0241791.s003.docx]

**S1 Table Correlation coefficient matrix of mobile variables and FIS measures in Nepal**

|  | Mobile phone ownership | Mobile phone expenditure | FIS score |
| --- | --- | --- | --- |
|  |  |  |  |
| Mobile phone ownership |  |  |  |
| Mobile phone expenditure | 0.665*** |  |  |
| FIS score | -0.619*** | -0.342*** |  |
| FIS prevalence | -0.583*** | -0.349*** | 0.902*** |

Note: values are Pearson correlation coefficients, based on the pooled sample comprising observations from across four panel rounds. *** p<0.001.
